# Extra-Uterine Pregnancy

**Published:** 1872-08

**Authors:** W. A. Matthews

**Affiliations:** Fort-Valley, Georgia


### EXTRA-UTERINE PREGNANCY.

BY W. A. MATTHEWS, M.D., FORT-VALLEY, GEORGIA.

The rather rare occurrence of extra-uterine pregnancy, and the difficulty of diagnosis, induce me to give a brief report of the following case that came under my observation during the present year:

Sylvia, a colored woman, aged about twenty-six years — the mother of two children, the youngest six years old — presented symptoms of pregnancy in July last, and the latter part of September, was noticeably enlarged. About the 1st of October, being greatly shocked by witnessing the stabbing of a man on the plantation where she was living, she commenced reducing in size, and all indications of pregnancy disappeared.

In November, indications of pregnancy again existed; about the 25th of the month, she was attacked with symptoms threatening abortion, for which laudanum was given, and relief afforded. During the months of December and January, she had frequent returns of similar attacks, and came under the care of Dr. W. L. Jones, an intelligent practitioner of Crawford county, who was repeatedly called to see her when thus troubled.

About the 28th of January, I visited Sylvia the first time; learned she had been in great pain, seemingly "hard labor," but was nearly easy when I arrived. On examination, I found the os firmly closed, and great tenderness. On placing my hand over the pubic region, I detected evident enlargement, such as indicated at least her fourth month of utero-gestation, and extreme soreness. The enlargement, she said, commenced in the left side. On interrogating the woman, she informed me that she first felt the movement of the fœtus about Christmas, and frequently since. The tenderness over the inter-uterine region being so great, I prescribed a blister, which was followed by prompt relief. She had tolerable health until about the 1st of March, pains occasionally recurring—sometimes ceasing; at other times, anodynes were given.

I heard nothing more from the case, directly, until the 17th day of June, when I received a note from Dr. Jones requesting me to see the woman with him. I went immediately as requested. Found her quiet, all pains having ceased. The Doctor informed me he found her, on his arrival, in much pain, indicating very active labor, though no impression whatever had been made upon the os uteri, which he could just touch, it being very high up. By request, I examined her, and found her condition as represented—os closed perfectly, and seemingly *unusually* hard. Enjoining quietude, we left her.

On the —— day of June, I was again summoned to see the woman (Dr. Jones being sick), and it not being convenient for me to see her, I requested my friend and partner, Dr. W. J.

Greene, to visit her. Dr. Greene found the pains to have ceased, which had been *very* violent, and the woman perfectly quiet. The Doctor instituted an examination; thought he passed his finger into the cavity of the uterus, and expresses the opinion that it (the womb) contained nothing, which opinion was correct, as the sequel proved.

Three days thereafter, I was again sent for, the pains having returned with unusual violence. I found her quiet, all pains having ceased. Wishing to see the patient when under the influence of uterine contractions, I remained all night, and about two o'clock in the morning, was notified the pains had returned. I went immediately to her house, and during a most violent paroxysm of pain, examined the os, but found no impression was made by the contraction, although so strong. I also examined the abdomen during a paroxysm, which presented a rather peculiar appearance, being flattened on each side, and very prominent along the median line. My examinations, with the history given by Sylvia of her condition during her last pregnancy, etc., induced me to believe that there was probably induration, from some cause, of the *inner* os, preventing dilatation, as I thought I could distinctly detect the external os to be open and soft. The account given by Sylvia, above referred to, was to the effect that, during her pregnancy with her last child, she was, as she believed, in a similar condition—suffered much, and was for a long time under medical treatment; that on one occasion several physicians met in consultation on her case, and determined to open her abdomen; that while making preparations for the operation, one of the physicians proposed to defer it for a time, which was agreed to; and in four weeks, after an unusually hard labor of about fourteen hours, she was delivered of her now living child.

Three days subsequent to my last visit, with Drs. Greene and Jones, I again saw Slyvia. Found her in a high fever, with violent pains in her head, having had several convulsions. Bled her, and applied cold water to the head, which afforded relief. No change in the condition of the uterus. We prescribed rest, and small doses of tartrate of antimony to keep down excitement and cause relaxation.

Dr. Jones saw the case every day or two. There was no material change nor return of pains until the 24th June, about one week subsequent to my last visit, when she was again attacked with pains, the last of which, as I was informed by an intelligent lady who was present, continued forty minutes, without any *seeming* relaxation. During this pain, a noise was distinctly heard by all present, as of "something bursting" in the abdomen; felt also by the sufferer. The pains ceased, and she had no more. Within a few hours, she commenced complaining of abdominal tenderness, which increased rapidly until the time of her death, four days thereafter.

About eighteen hours after her death, in presence of Drs. Greene and Jones, I opened the abdomen carefully. A large quantity of bloody water gushed out, with some coagula. The fœtus—a *very* plump, well-developed female child—was found lying rather obliquely with its face to the right side, in contact with the liver, its breech to the symphysis, the umbilical cord passing over the body. After severing the cord and removing the fœtus, I traced the cord to the placenta, which was found in the side near the left ovary, attached to the Fallopian tube and adjacent parts. The Fallopian tube seemed to have been lacerated obliquely, and presented a very dark appearance, as did also the omentum. The uterus was then examined and removed, and found to be empty, as Dr. Greene supposed. It was about as large as the head of a small fœtus, and presented a rather peculiar, shapeless appearance—of a dull, whitish color.

The examination was not as thorough as I have since wished it had been, but such as to present the above appearances. It was clearly a case of Fallopian pregnancy, the fœtus, with the "waters," having been discharged into the cavity of the abdomen by the laceration of the tube during the protracted pain alluded to, causing peritonitis, which terminated the patient's life. As to the question when conception took place, whether nine or twelve months before death, we can not tell. A better-developed fœtus I never saw; weighed about eight and a half or nine pounds, and remained alive until the day of the mother's death. Could we have been certain that the fœtus was

*extra-uterine*, it might have been saved by abdominal section; remotely possibly, the mother also. It presented the only hope.

As such cases are rare, well-knowing the difficulties that beset the path of a conscientious physician, and hoping that light may be thrown upon some similar case, I send this to you for publication. Many interesting features have been omitted, for fear of encroaching upon your valuable space.

